# Efficacy of proteolytic enzyme bromelain on health outcomes after third molar surgery. Systematic review and meta-analysis of randomized clinical trials

**DOI:** 10.4317/medoral.22731

**Published:** 2018-12-24

**Authors:** Mário-Luis-Tavares Mendes, Edmundo-Marques do Nascimento-Júnior, Daniele-Machado Reinheimer, Paulo-Ricardo-Saquete Martins-Filho

**Affiliations:** 1 MSc, Investigative Pathology Laboratory, Federal University of Sergipe, Brazil; 2 PhD, Investigative Pathology Laboratory, Federal University of Sergipe, Brazil

## Abstract

**Background:**

Bromelain is a cysteine protease isolated from pineapple with a range of biological properties including platelet aggregation inhibition and anti-inflammatory effects. Recent studies have evaluated the clinical implications of bromelain in reducing postoperative inflammatory complications after third molar surgery, but the results are contrasting. This systematic review and meta-analysis evaluated the effects of bromelain on health outcomes in patients submitted to third molar surgery.

**Material and Methods:**

The study was conducted following the PRISMA statement. Searches were conducted in six electronic databases and Google Scholar from inception to May 2018. The following elements were used to define eligibility criteria: (1) population: patients undergoing third molar surgery; (2) intervention and controls: bromelain vs placebo or no-treatment control group; (3) outcomes: quality of life, postoperative pain, rescue analgesic consumption, facial swelling, and trismus; and (4) study type: randomized clinical trials (RCTs). Treatment effects were defined as weighted (WMD) or standardized mean difference (SMD) and 95%CIs.

**Results:**

Six RCTs were included in the meta-analysis. There was large effect size of bromelain on improving physical appearance (SMD -0.77, CI% 95 -1.11 to -0.42), social isolation (SMD -0.97, CI% 95 -1.74 to -0.21), and sleep quality (SMD -1.19, CI% 95 -1.97 to -0.40) during the first postoperative week. Differences in pain intensity were found during the first 24h (SMD -0.49, CI 95% -0.82 to -0.17) and 7 days after surgery (SMD -0.52, CI 95% -0.79 to -0.24). No evidence was found that bromelain was effective in reducing trismus and facial swelling.

**Conclusions:**

The currently available evidence suggests that bromelain has a beneficial effect in reducing pain and has a positive impact on patient quality of life after third molar surgery. However, therapeutic advances for the use of bromelain need a high level of evidence and further head-to-head RCTs are needed to inform clinical choices.

** Key words:**Bromelain, third molar, oral surgical procedures.

## Introduction

In recent years, evidence has emerged on the efficacy of proteolytic enzymes in diverse health-related conditions. Bromelain is a complex natural mixture of protein-digesting enzymes derived from the fruit or stem of pineapple (Ananas cosmosus) used as a phytomedical compound with a range of therapeutic benefits (1). It has diverse biological properties including platelet aggregation inhibition and anti-inflammatory effects which seem to be related to proteolytic activity (2). In addition, it has been suggested that aqueous extract from the crown leaves of pineapple containing bromelain presents antibacterial and antifungal activities, and presents potential use in treating microbial infections (3).

Bromelain is considered to be nontoxic and may be used at daily doses of 200 to 2,000 mg/kg, for prolonged periods of time (4). The degree to which bromelain and its components are absorbed and retain function still remains to be elucidated, but studies have suggested that oral administration of this proteolytically active pineapple extract is absorbed into the intestines and remains biologically active with a half-life of ~6–9 h and plasma concentration reaching as much as 5,000 pg/ml by 48 h after oral multidosing of 3g/day (5).

Reports from preliminary clinical studies have indicate the potential safety and efficacy of bromelain-based enzymatic debridement in chronic wounds(6) and deep burn injuries (7). In addition, reports have shown that anti-inflammatory and analgesic characteristics of bromelain could be useful in the treatment of several chronic inflammatory disorders as osteoarthritis and rheumatoid arthritis (8–10). Recent studies have evaluated the clinical implications of bromelain in reducing postoperative inflammatory complications after third molar surgery (11,12), but the results are contrasting (13).

Removal of impacted third molars is one of the most frequent procedures in oral surgery, but is commonly associated to postoperative pain, swelling, and trismus (14). These complications are thought to arise from inflammatory response which is a direct and immediate consequence of the surgical procedure, and may lead in patient discomfort and negatively affect their quality of life (15). The aim of this study is to perform a systematic review and meta-analysis to evaluate the effects of bromelain on postoperative pain, analgesic consumption, facial swelling, trismus, and quality of life in patients submitted to third molar surgery.

## Material and Methods

This study was conducted following the Preferred Reporting Items for Systematic Reviews and Meta-Analyses statement(16) and supplemented by guidance from the Cochrane Collaboration Handbook for Systematic Reviews of Interventions (17). Institutional review board approval and informed consent were not required for this systematic review and meta-analysis.

-Search Strategy

Searches for RCTs were performed in PubMed, Web of Science, SCOPUS, Cochrane Central Register of Controlled Trials, and the website ClinicalTrials.gov from inception to May 2018. A gray-literature search included Google Scholar and OpenThesis. The first 100 results of the Google Scholar search were analyzed. The search was limited to studies published in full-text versions, without language restriction. The reference lists of all eligible studies and reviews were scanned to identify additional studies for inclusion. The structured search strategy used the following terms: (proteolytic enzyme OR protease OR proteinase OR bromelin OR bromelain) AND (third molar OR third molars OR wisdom tooth OR wisdom teeth). To expand the number of eligible articles, there is no use of filters in the search.

-Study Selection and Eligibility Criteria 

Two reviewers (M.L.T.M. and E.M. do N.-J.) independently screened the search results and identified studies that were potentially relevant based on their title and abstract. Relevant studies were read in full text and selected according to eligibility criteria. Disagreements between the 2 reviewers were resolved by consensus or by a third reviewer (P.R.S.M.-F.).

The following PICOT (Population, Intervention, Comparison, Outcomes, Type of study) elements were used to define the eligibility criteria: 1) population (patients submitted to removal of impacted third molars), 2) intervention and comparison (administration of bromelain vs placebo or no treatment control group), 3) outcomes (primary outcome was quality of life and secondary outcomes were postoperative pain, rescue analgesic consumption, facial swelling, and trismus), and 4) study type (RCTs). Eligible studies must report at least 1 of the outcomes of interest.

-Data Extraction and Risk of Bias Assessment

Using a standardized data extraction sheet, the following information from the studies were extracted: demographic characteristics of study participants, preoperative and postoperative medication, duration of follow-up, and outcome data.

Risk of bias was assessed according to the Cochrane guidelines for RCTs. Seven domains were assessed for evaluation: sequence generation and allocation concealment (selection bias), blinding of participants and personnel (performance bias), blinding of outcome assessment (detection bias), incomplete outcome data (attrition bias), selective outcome reporting (reporting bias), and other potential sources of bias. Risk of bias was rated as low, unclear, or high according to established criteria (17).

Data extraction and risk of bias assessment were performed by two independent reviewers (M.L.T.M. and E.M. do N.-J.), and disagreements were resolved by consensus or by a third reviewer (P.R.S.M.-F.).

-Data Synthesis

Treatment effects of bromelain on quality of life, pain, analgesic consumption, trismus, and facial swelling were defined as standardized mean difference (SMD) and 95% confidence intervals (CIs). The use of rescue medication during the first postoperative week was analyzed using weighted mean difference (WMD). To calculate the effect sizes, means and standard deviations (SD) were obtained for each study group and outcome of interest. Differences between groups were meta-analyzed using the generic inverse-variance method.

Effect size was determined by calculating Cohen’s d statistic (18). A value of 0.2 was considered a small effect, a value of 0.5 a medium effect, and a value of 0.8 a large effect. A negative effect size indicated that bromelain had beneficial effects on short-term outcomes. Trismus and facial swelling were analyzed based on change-from-baseline measures (19).

A forest plot was used to present the effect sizes and the 95% CIs. A 2-tailed p value < 0.05 was used to determine significance. Statistical heterogeneity was assessed using the Cochran Q test(20) and quantified by the I2 index (21). Subgroup analyses were performed according to the follow-up time. Leave-one-out sensitivity analysis was performed to evaluate the influence of control groups (placebo or no treatment control group) on effect sizes. Analyses were conducted using Review Manager, version 5.3 (Cochrane IMS).

-Grading the Strength of Evidence

We graded the strength of evidence for the effect of bromelain on quality of life and postoperative pain as high, moderate, low or very low using the Grading of Recommendations Assessment, Development, and Evaluation (GRADE) rating system. In the GRADE system, RCTs begin as high-quality evidence but may be lowered by 1 or more of 5 categories of limitations: risk of bias, inconsistency (heterogeneity), indirectness of evidence, imprecision, and publication bias (22,23).

## Results

-Data Sources 

**Figure 1:**
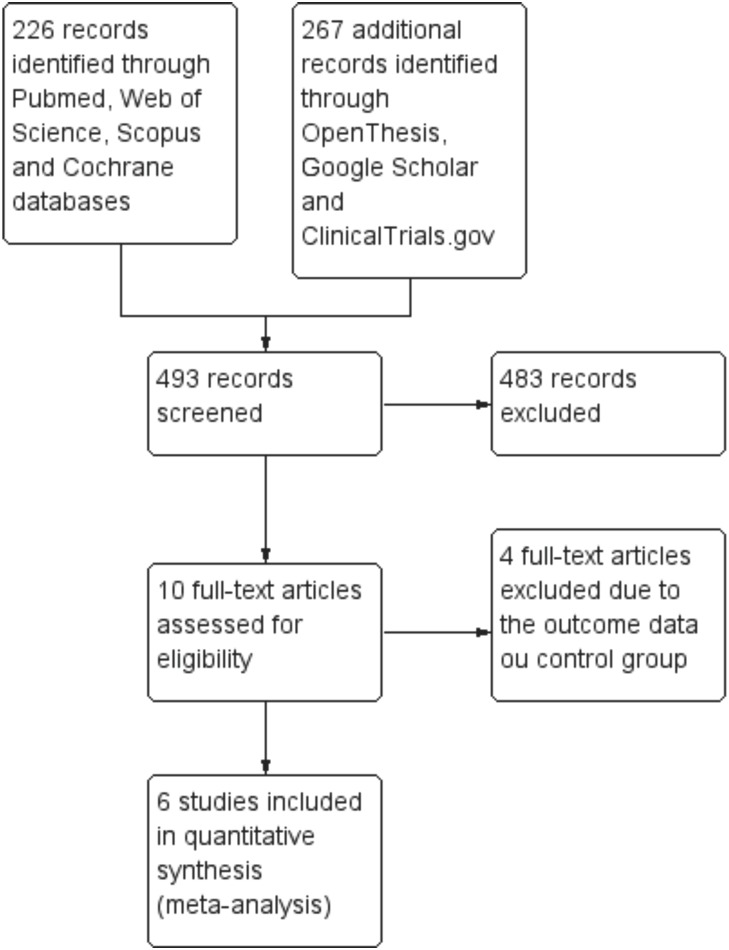
Flow diagram of literature search and screening process.

Search strategy yielded 493 potentially relevant studies. After screening titles and abstracts, 10 full-text articles were assessed for eligibility and 6 RCTs (11–13,24–26) were included in the meta-analysis. A flow diagram of the study selection process and specific reasons for exclusion are detailed in Figure 1.

-Study Characteristics and Risk of Bias Assessment

The total number of patients included in the RCTs was 312. Most surgical procedures were performed for removal impacted mandibular third molars in healthy young adults. In all studies, bromelain was administered orally, but there were differences in daily dose frequency and time of treatment. Paracetamol 500mg was prescribed as a rescue medication for pain relief in 4 studies (11,13,24,25) and analgesic consumption within the first postoperative week was included as an outcome of interest.

**Figure 2:**
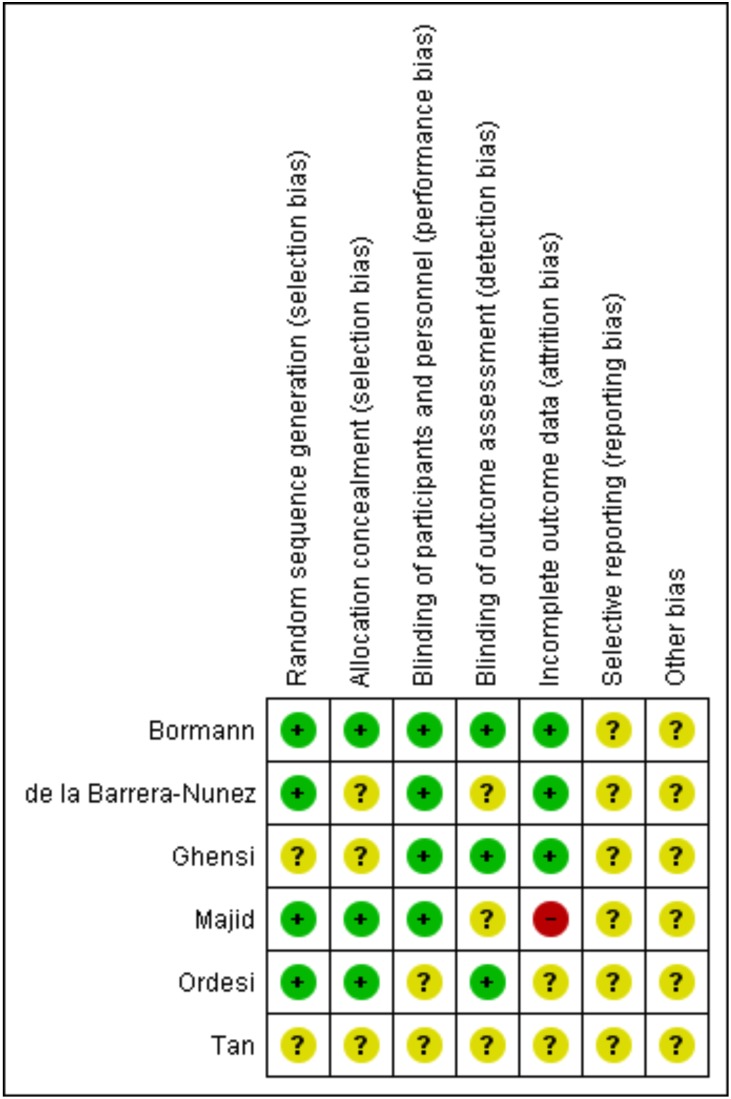
Risk of bias assessment. Footnote: (+) low risk of bias; (-) high risk of bias; (?) unclear risk of bias.

Postoperative pain was measured using a 10-point visual analogue scale (VAS)(11-13,25) or a 0-4 Likert-type scale (26). Trismus was evaluated as maximum interincisal distance (MID) (11,13,24). Measurements of postoperative swelling were heterogeneous among studies and included 3D evaluation (25), 10-point VAS (13), and use of facial linear distances (11,12,24). Three studies evaluated quality of life during the first postoperative week, 2 using the Postoperative Symptom Severity (PoSSe) scale (12,24) and one using the Majid scale (11). The main characteristics of RCTs are presented in  Table 1. Most studies had unclear risk of bias (Fig. 2).

**Table 1:**
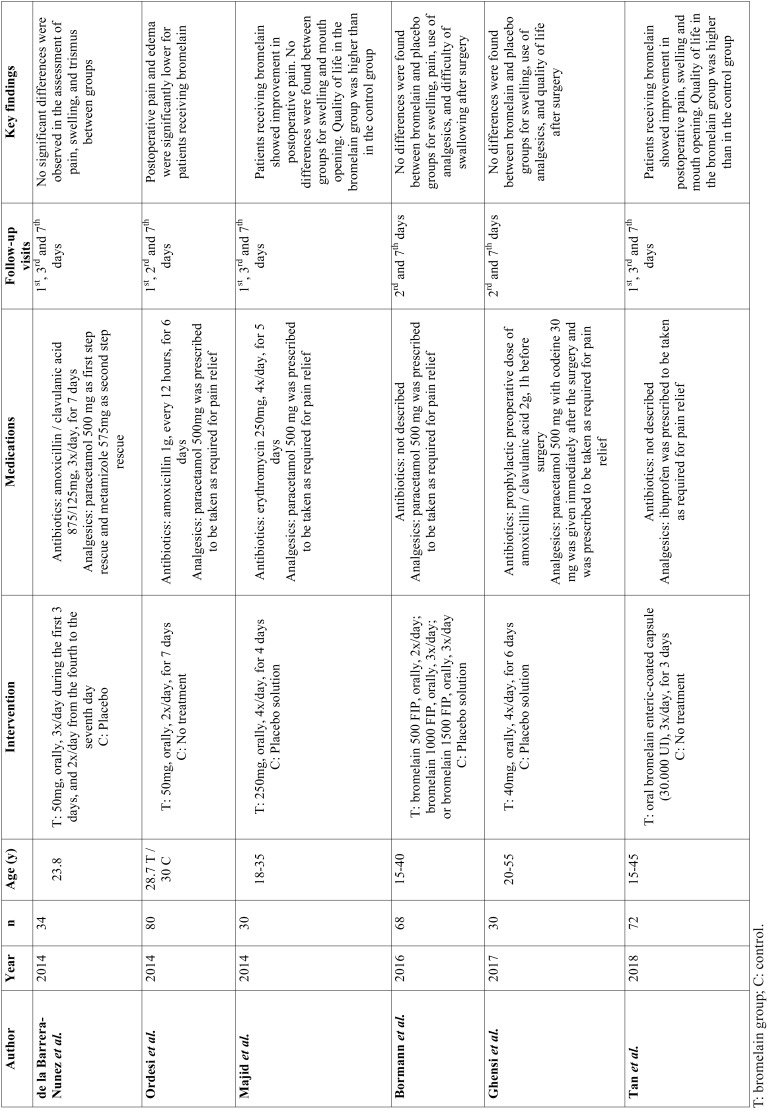
Characteristics of studies included in the meta-analysis.

-Data Synthesis

Quality of life

**Figure 3:**
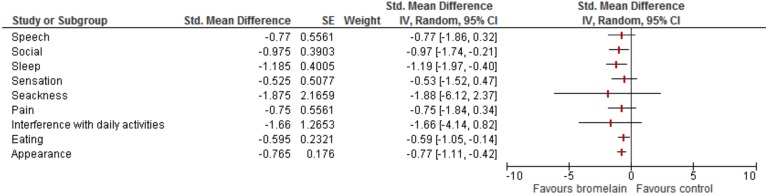
Forest plot showing the effect of bromelain on quality of life after third molar surgery.

The meta-analysis evaluating the effect of bromelain on quality of life after third molar removal was based on results of 3 studies (11,12,24). It was found a moderate to large effect size of bromelain on improving eating (SMD -0.59, CI% 95 -1.05 to -0.14), physical appearance (SMD -0.77, CI% 95 -1.11 to -0.42), social isolation (SMD -0.97, CI% 95 -1.74 to -0.21), and sleep quality (SMD -1.19, CI% 95 -1.97 to -0.40) during the first postoperative week (Fig. 3).

-Postoperative pain

**Figure 4:**
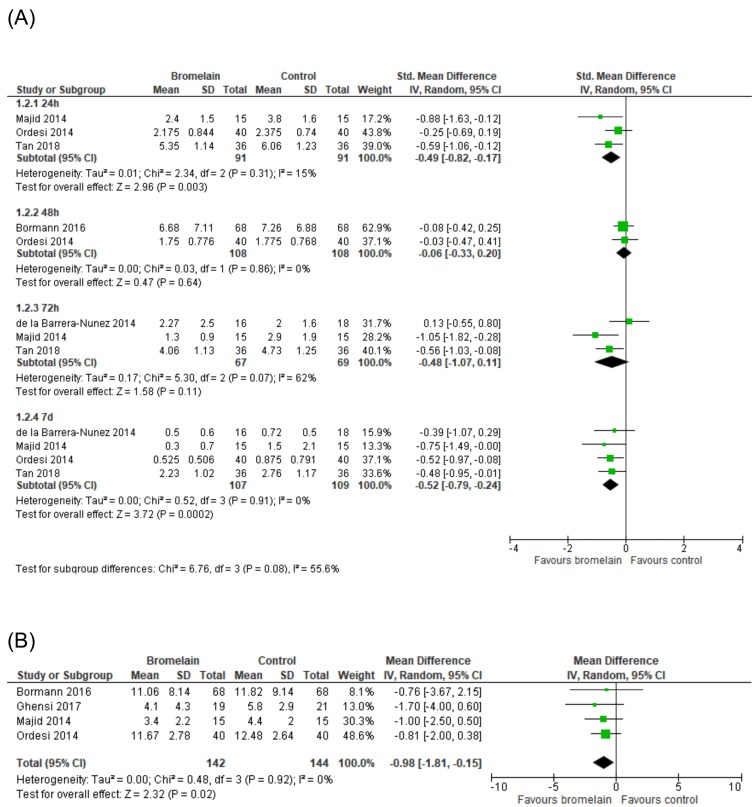
Efficacy of bromelain on pain (A) and analgesic consumption (B) within the first postoperative week.

Five RCTs (11–13,25,26) included in this meta-analysis provided sufficient data for pain evaluation during the first postoperative week. We found a moderate effect size of bromelain in reducing pain. Differences in pain intensity were found during the first 24h (SMD -0.49, CI 95% -0.82 to -0.17, *p* = 0.003, I2 = 15%) and 7 days after surgery (SMD -0.52, CI 95% -0.79 to -0.24, *p* < 0.001, I2 = 0%) (Fig. 4).

-Analgesic consumption

Data on analgesic consumption during the first postoperative week was extracted from 4 RCTs(11,24–26). A reduction in rescue medication was found among patients using bromelain compared with control group (WMD -0.98, CI 95% -1.81 to -0.15, *p* = 0.02, I2 = 0%) (Fig. 4).

-Trismus and facial swelling 

**Figure 5:**
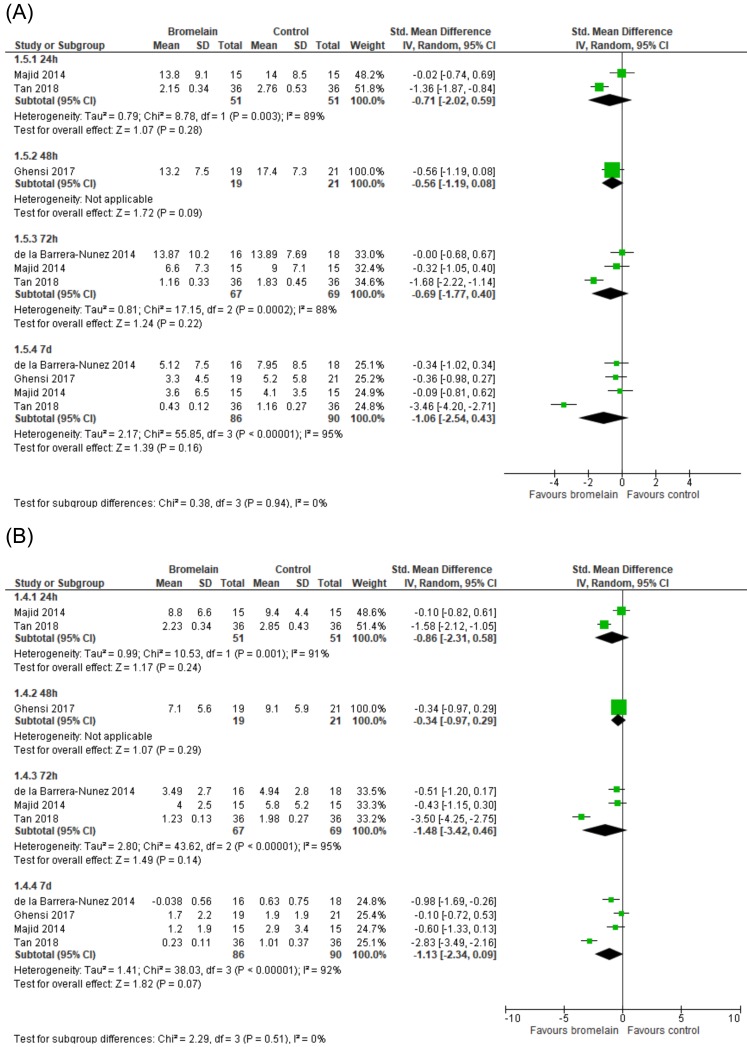
Efficacy of bromelain on trismus (A) and facial swelling (B) within the first postoperative week.

Four RCTs(11–13,24) included in these meta-analyses provided sufficient information to analyze the effects of bromelain on trismus and facial swelling. No evidence was found that bromelain was effective in reducing trismus (Fig. 5) and facial swelling (Fig. 5) following third molar surgery.

-Sensitivity analysis

To investigate the potential influence of control groups on the overall meta-analysis estimation, we omitted one study at a time. The “leave-one-out” analysis showed that effect sizes did not change substantially with the exclusion of any one study.

-Strength of evidence

We graded the effects of bromelain on quality of life and pain in patients submitted to third molar surgery as moderate quality of evidence as per the GRADE criteria ( Table 2).

**Table 2:**
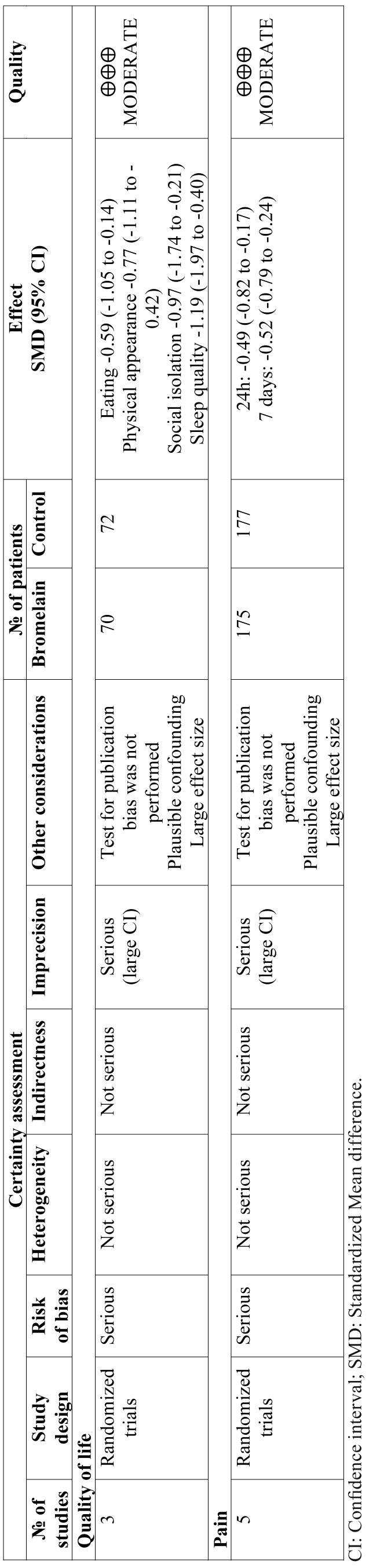
GRADE evidence profile for efficacy of bromelain on health outcomes in third molar surgery.

## Discussion

Nonsteroidal anti-inflammatory drugs (NSAIDs) are commonly used in the management of short-term outcomes following third molar surgery (27). Although NSAIDs are effective for postoperative pain control, gastrointestinal consequences of NSAIDs are significant and need to be considered when prescribing this group of medications to patients (28). The risk of adverse events with traditional NSAIDs has led to the development of alternative therapeutic options. Important anti-inflammatory response without side effects have been shown using autologous biomaterial (29), low-level laser therapy (30), and phytotherapy (26). Bromelain has been indicated as a natural alternative to conventional treatment with NSAIDs. In this systematic review and meta-analysis, we evaluated the efficacy of proteolytic enzyme bromelain in reducing postoperative inflammatory complications and its effects on quality of life in patients submitted to third molar surgery.

In this study, we showed that bromelain had a moderate effect size in reducing pain during the first 24h and 7 days after surgery and provided a reduction in the average number of rescue medication required per patient. Although no evidence was found that bromelain was effective in reducing trismus and facial swelling, bromelain had a moderate to large effect size on improving several domains in quality of life (eating, physical appearance, social isolation, and sleep quality) during the first postoperative week.

It has been shown that bromelain has anti-inflammatory effects due to the inactivation of bradykinin in inflamed tissues leading in decreased levels of prostaglandin E2 (PGE2) and substance P (31,32). In addition, bromelain seems to play an important role as plasminogen activator leading in decreased levels of serum plasmin and increased vascular permeability, which allows the edema fluid to reenter the vessels and resolving stasis (33). These physiological effects can lead to significant reductions in pain and swelling while enhancing circulation to the site of surgery. Although reducing postoperative pain and improved in quality of life are achieved with bromelain, the results on decreasing facial swelling following third molar surgery are still inconclusive. Furthermore, there is striking lack of safety data for bromelain and rigorous studies are needed to fully characterize bromelain as therapeutic anti-inflammatory agent in surgical care.

The results of this meta-analysis should be interpreted with caution because the pharmacological activities of bromelain in each study and effect estimates may have been influenced by a range of factors including the number of active ingredients whose ratio to each other might vary according to soil composition, climatic conditions during plant growth, variety of pineapple, and manufacturing process (2). In addition, despite commercially available chemical and nutraceutical preparations of bromelain contain predominately stem bromelain (34), the method of purification and the source of pineapple extract in some studies included in this review is uncertain. It has been found that stem bromelain contains high quantities of protease content when compared with bromelain derived from the fruit (5,35).

Moreover, the studies evaluated in this review co-prescribed the bromelain along with rescue analgesics which may lead to confound drug-effect associations. Interestingly, although we found a reduction of pain in patients using bromelain, there was a mean decrease of only one tablet of analgesic rescue medication during the first postoperative week. Pain relief is a subjective experience and the results observed for patients using bromelain could be explained in part by the placebo effect. Optimization of drug prescription and medication management after surgeries should be an important part of clinical decision making but results from head-to-head trials comparing the anti-inflammatory effect of bromelain with and without NSAIDs/analgesics after third molar surgery are scarce and a pragmatic recommendation of bromelain cannot be supported.

The currently available evidence suggests that bromelain has a beneficial effect in reducing pain and has a positive impact on patient quality of life after third molar surgery. However, therapeutic advances for the use of bromelain need a high level of evidence and further head-to-head RCTs are needed to inform clinical choices.
